# Supplementary material for: DIRAS3 Inhibits Ovarian Cancer Cell Growth by Blocking the Fibronectin-Mediated Integrin β1/FAK/AKT Signaling Pathway
Source: Cells. 2025 Aug 13;14(16):1250. doi: 10.3390/cells14161250 (PMC12384585; doi:10.3390/cells14161250)
Supplement: Supplementary file 1 [file cells-14-01250-s001.zip › cells-3689714-supplementary.pdf]

Supplemental Figure S1

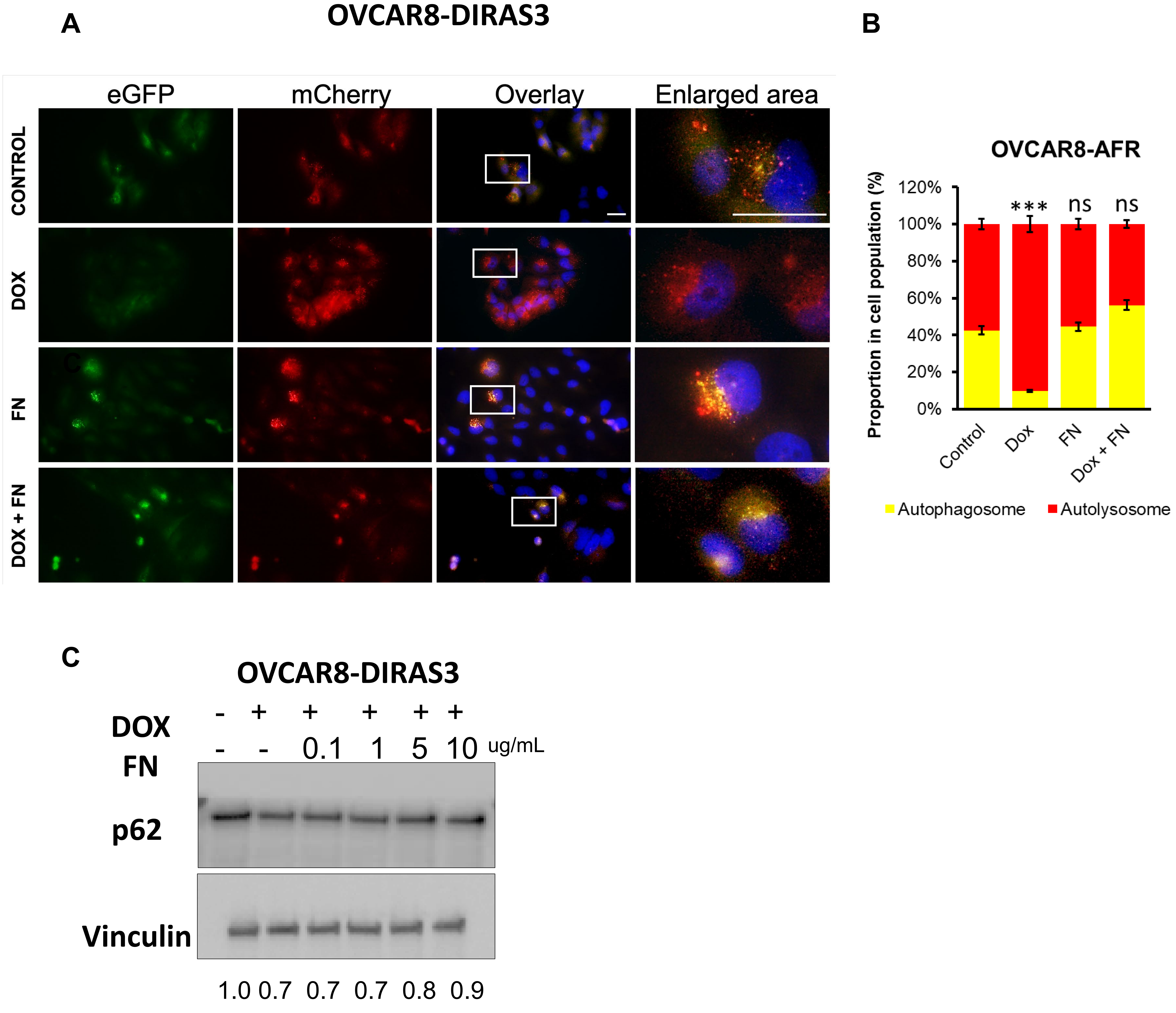

Supplemental Figure S1. (A-B)) Measurement of autophagic flux. Representative images and signal quantifications are presented from the analysis of 3 separate high-power field images (scale bar: 20 μm). Statistical analysis was performed with one-way ANOVA; ns: $p > 0.05$, ***$p < 0.001$ compared to control. Error bars represent SD. **(C)** Western blot analysis was performed to examine DIRAS3 and LC3 expression. SKOV3-DIRAS3 and OVCAR8-DIRAS3 cancer-cells were seeded at 1.5×10^5 cells/well and treated with DOX (1μg/mL) with or without fibronectin (FN) (0.1-10 ug/mL). Densitometry was performed with ImageJ.

Supplemental Figure S2

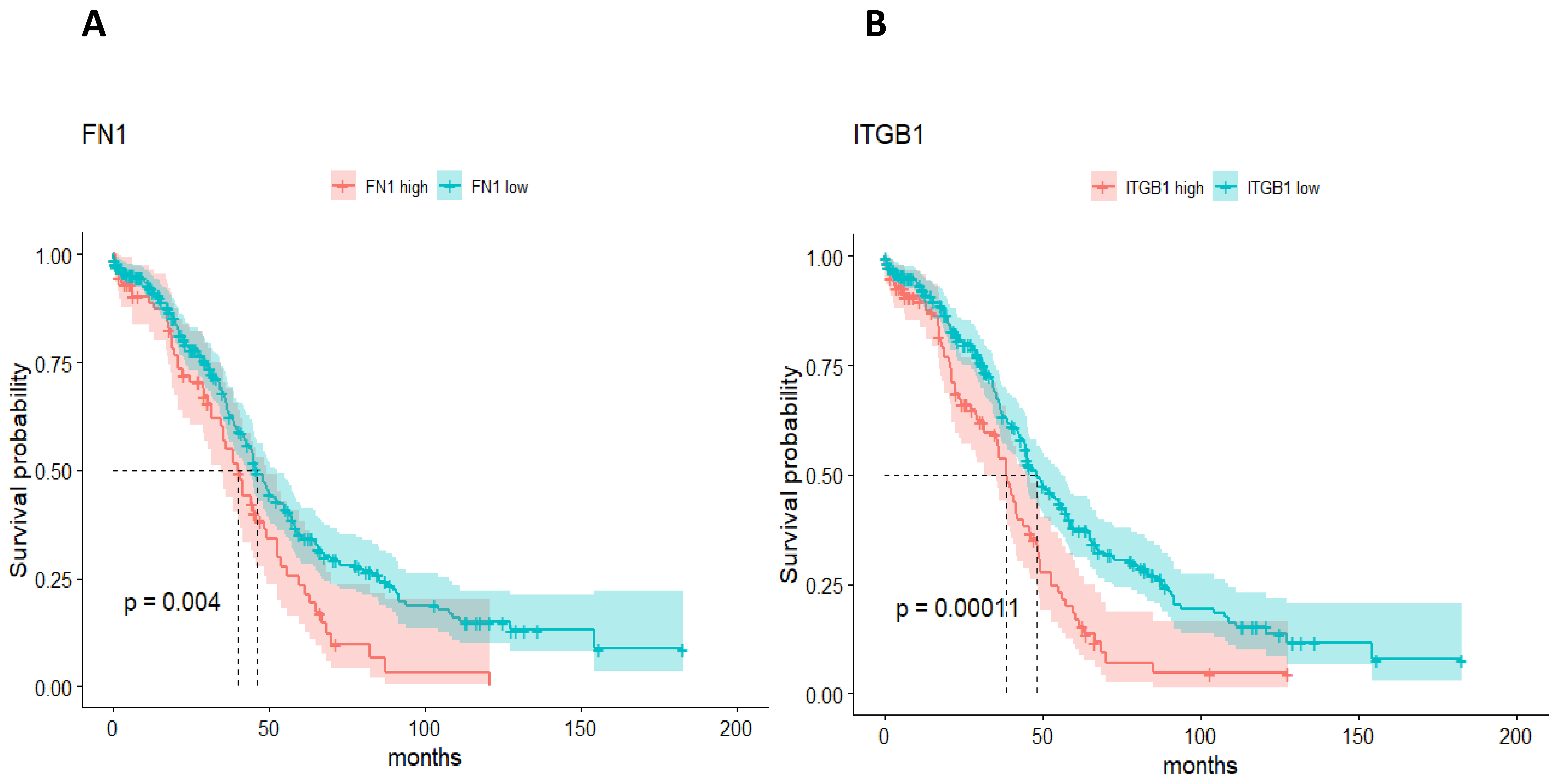

Supplementary Figure S2. Fibronectin and integrin-β1 are correlated with ovarian cancer survival rate. To evaluate the clinical prognostic relevance of FN1 and ITGB1 gene expression in ovarian cancer, survival analysis was performed using data from The Human Protein Atlas. Patients were stratified into high or low expression groups. Kaplan-Meier survival curves were generated and the difference in overall survival was assessed. For statistical analysis, we used the log-rank test. Results showed that high FN was associated with poor overall survival (log-rank p=0.004). Similarly, high levels of ITGB1 was correlated with decreased survival (log-rank p=0.00011).
